# Metamorphosis by ATG13 and ATG101 in human autophagy initiation

**DOI:** 10.1080/15548627.2023.2230054

**Published:** 2023-07-02

**Authors:** Anoshi Patel, Alex C. Faesen

**Affiliations:** Max-Planck Institute for Multidisciplinary Sciences, Laboratory of Biochemistry of Signal Dynamics, Göttingen, Germany

**Keywords:** metamorphosis, ATG9A, ATG2A, lipid transfer, membrane contact site, super-complex

## Abstract

Autophagosome biogenesis requires the timely assembly of a complex machinery. However, it is unclear how a putative stable super-complex is assembled and disassembled, and how the different parts cooperate to perform its overall function. To shed light on these questions, we embarked on an effort to biochemically reconstitute the human initiation complexes *in vitro* with purified full-length components. This approach revealed that all initiation subcomplexes together form a stable super-complex on an interaction platform consisting of ATG9A, ATG13 and ATG101. An unusual metamorphosis of ATG13 and ATG101 creates a rate-limiting step in the assembly of the ATG9A-ATG13-ATG101 complex. Furthermore, the interaction of ATG2A with the ATG9A-ATG13-ATG101 complex and WIPI4 cooperatively enhances both the vesicle tethering and lipid transfer of this complex. It seems likely that the spontaneous self-assembly of the super-complex is inhibited before autophagy initiation, a mechanism where the rate-limiting metamorphosis of ATG13 and ATG101 could play a central regulatory role.

**Abbreviations:**

ATG, Autophagy-related, HORMA, protein domain named after HOP1-MAD2-REV7; RB1CC1, RB1 inducible coiled-coil 1; ULK, Unc-51-like kinase

*De novo* autophagosome biogenesis is a complex process. It requires the coordinated interplay of numerous autophagy-related (ATG) proteins, which co-localize when macro-autophagy, hereafter autophagy, is initiated. In our recent work [1], we explored a hypothetical mechanism that could regulate the assembly of a putative initiation super-complex consisting of the various functional subcomplexes. The initiation of autophagy requires the ULK (Unc-51-like kinase) kinase complex (consisting of ULK1 or ULK2 kinase, RB1CC1 (RB1 inducible coiled-coil 1)/FIP200, ATG13 and ATG101), ATG9A-positive vesicles, the PI3-kinase complex (PIK3C3 (phosphatidylinositol 3-kinase catalytic subunit type 3)/VPS34, PIK3R4 (phosphoinositide-3-kinase regulatory subunit 4)/VPS15, ATG14 and BECN1 (Beclin 1)) and a lipid transfer complex (ATG2A with the PI3P-binding WIPI4 adaptor protein). We were particularly intrigued by the presence of ATG13 and ATG101 within this machinery. These HORMA domain-containing proteins (named after the HOP1, REV7 and MAD2 proteins) are part of a family of unusual scaffolding proteins. Aided by their unusual energy landscape, the purposely slow spontaneous metamorphosis of HORMA domains serves as a regulatory switch that dictates the assembly rate of (large) effector complexes. Like other HORMA domain proteins, ATG13 and ATG101 are ideally placed to function as a “molecular switch” to trigger the assembly of super-complexes. This hypothesis, however, was untested.

To test for the existence of a putative super-complex, the first challenge was to purify recombinant full-length versions of all potential components of this super-complex. When mixing the purified proteins, we indeed observed that all self-assembled in a single super-complex containing ULK1, RB1CC1, ATG13, ATG101, ATG9A, ATG14 and BECN1. PIK3R4 and PIK3C3 were not part of this reconstitution. Interestingly, the super-complex did not form in the absence of the subcomplex ATG9A-ATG13-ATG101, providing the first hint of the coordinating role of ATG13 and ATG101.

Are ATG13 and ATG101 however metamorphic proteins? Metamorphosis is an unusual ability of proteins to switch between two topologically distinct folds or conformers. Each conformer has a different three-dimensional structure and typically one conformer interacts with the client protein to trigger the assembly of complexes. The emerging paradigm for HORMA domain-containing proteins is that they default to a distinct conformer, before converting to a second conformer accompanied by an absolute change in interaction spectrum. This change in structure allowed us to purify two ATG13 conformers, which each interacted with either ATG101 or the N-terminus of ATG9A (first 60 residues). Since considerable activation energy needs to be invested to induce the structural changes in the HORMA domain topology, metamorphosis is typically slow. Indeed, both ATG13 and ATG101 default to an inactive non-ATG9A-binding state and metamorphosis is obligatory before interacting with ATG9A (Figure 1). As seen with other HORMA domain-containing proteins, the dimerization of ATG13 and ATG101 dramatically accelerated complex formation from 18–24 h to 30 min. Mutants that lack specific metamorphic elements, i.e., ATG13^Δseatbelt^ or ATG101^ΔN^, could not rescue the loss of autophagic flux in *atg13*^*-/-*^ or *atg101*^*-/-*^ knockout murine embryonic fibroblasts, nor the perturbed co-localization of ATG9A and ATG13. Additionally, these mutants prevented the co-localization of ATG16L1, RB1CC1, ATG14 and WIPI2 under autophagy inducing conditions. Collectively, these observations suggest that the obligatory and rate-limiting metamorphosis of ATG13 and ATG101 is involved in the regulation of the early steps of autophagy initiation.

**Figure 1.**
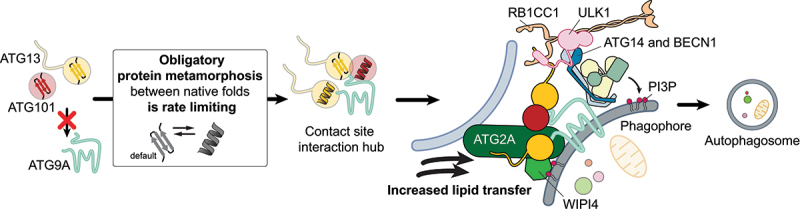
Metamorphosis of ATG13 and ATG101 in human autophagy initiation. the assembly of the ATG9A-ATG13-ATG101 subcomplex requires the metamorphosis of ATG13 and ATG101. Since this metamorphosis is slow, this unusual mechanism introduces a rate-limiting step in the assembly of the ATG9-ATG13-ATG101 complex. Once formed, the ATG9A-ATG13-ATG101 complex forms the interaction hub for the recruitment of all autophagy initiation subcomplexes to create a stable super-complex. The interaction of ATG2A with the ATG9A-ATG13-ATG101 and WIPI4 cooperatively enhances both its vesicle tethering and lipid transfer activities.

The fundamental question of how lipids are transported unidirectionally from the ER to the phagophore remains unanswered. It is currently unclear what defines the minimal “unit” that, if isolated or reconstituted in homogenous form, retains the molecular properties expected of the physiological membrane contact sites between the ER and the phagophore. The membrane contact site would contain a membrane tether, a role which is likely taken up by the ATG2 lipid transfer proteins with additional factors and adaptor proteins, like WIPI4. We found that the ATG9A-ATG13-ATG101 complex also forms a stable complex with ATG2A-WIPI4. Using established assays, we could show that ATG9A indeed accelerates lipid transfer by ATG2A, presumably by providing lipid scramblase activity. To our surprise, we observed an additional increase of lipid transfer by ATG2A when ATG9A was bound to ATG13 and ATG101. The mechanism behind this acceleration is unclear. We observed that vesicle tethering and lipid transfer activities by ATG2A alone is a relatively weak, but both activities were progressively enhanced with the addition of WIPI4 (when phosphatidylinositol 3-phosphate (PI3P) was present in the vesicle), ATG9A and ATG13-ATG101. This suggests that ATG2A serves as a functional “co-incidence sensor” for the successful assembly of the super-complex, as its activity requires the cooperative association and activity from all canonical autophagy initiation subcomplexes. This could provide a way of “sensing” the co-incidence of early autophagy initiation factors as a mature membrane contact site before committing to autophagosome formation.

Overall, our study takes a first step in shedding light on the metamorphic behavior of ATG13 and ATG101, and its implications on autophagy initiation. While many requisite features of HORMA-based signaling have been identified in ATG13 and ATG101, we are only beginning to understand how these are involved in regulating the initiation of autophagy.
